# Metagenomic Next-Generation Sequencing vs. Traditional Microbiological Tests for Diagnosing Varicella-Zoster Virus Central Nervous System Infection

**DOI:** 10.3389/fpubh.2021.738412

**Published:** 2022-01-21

**Authors:** Yunqi Zhu, Miaomiao Xu, Chengyuan Ding, Zhihang Peng, Weixiao Wang, Binghu Sun, Jian Cheng, Chen Chen, Wei Chen, Hongxia Wei, Zhiliang Hu

**Affiliations:** ^1^Department of Infectious Disease, The Second Hospital of Nanjing, Nanjing University of Chinese Medicine, Nanjing, China; ^2^Center for Global Health, School of Public Health, Nanjing Medical University, Nanjing, China; ^3^School of Public Health, Nanjing Medical University, Nanjing, China; ^4^Department of Clinical Research Center, The Second Hospital of Nanjing, Nanjing University of Chinese Medicine, Nanjing, China

**Keywords:** metagenomic next-generation sequencing, varicella-zoster virus, central nervous system infection, polymerase chain reaction, nucleic acid test

## Abstract

**Background:**

Unbiased metagenomic next-generation sequencing (mNGS) detects pathogens in a target-independent manner. It is not well-understood whether mNGS has comparable sensitivity to target-dependent nucleic acid test for pathogen identification.

**Methods:**

This study included 31 patients with chickenpox and neurological symptoms for screening of possible varicella-zoster virus (VZV) central nervous system (CNS) infection. Microbiological diagnosing of VZV cerebrospinal fluid (CSF) infection was performed on stored CSF samples using mNGS, quantitative and qualitative VZV-specific PCR assays, and VZV IgM antibodies test.

**Results:**

The median age was 30.0 [interquartile range (IQR), 24.3–33.3] years. 51.6% of the patients were men. About 80.6% of the patients had normal CSF white blood cell counts (≤ 5 × 10^6^/L). VZV IgM antibodies presented in 16.1% of the CSF samples, and nucleic acids were detectable in 16.1 and 9.7% using two different VZV-specific real-time PCR protocols. Intriguingly, maximal identification of VZV elements was achieved by CSF mNGS (*p* = 0.001 and *p* = 007; compared with qualitative PCR and VZV IgM antibody test, respectively), with sequence reads of VZV being reported in 51.6% (16/31) of the CSF samples. All VZV PCR positive samples were positive when analyzed by mNGS. Of note, human betaherpesvirus 6A with clinical significance was unexpectedly detected in one CSF sample.

**Conclusions:**

Our study suggests that CSF mNGS may have higher sensitivity for VZV detection than CSF VZV PCR and antibody tests, and has the advantage of identifying unexpected pathogens.

## Introduction

Central nervous system (CNS) infections have led to a substantial morbidity and mortality in clinical practice; however, the etiologic agents remain undetermined in around half of the cases (1, 2). The diagnostic challenge is that more than 100 infectious agents can cause encephalitis, and that the initial neurological manifestations of many CNS infections are non-specific (3–5). It is difficult to measure all the CNS pathogens by traditional microbiological tests, such as morphological, serological, molecular, and culture methods (5). An acceptable, but not optimal, strategy is to target the most possibly causative pathogens based on a clinical judgment. However, in this context, the efficacy of pathogen discovery would largely depend on the clinical experience of the treating physician. Moreover, the etiologies of cases with atypical manifestations or caused by rare pathogens are likely to be unconfirmed.

Unlike the traditional pathogen-detection methods, which require a prior knowledge of the causative agents, the unbiased metagenomic next-generation sequencing (mNGS) identifies pathogens in a target-independent way (6). It has been reported that cerebrospinal fluid (CSF) mNGS helps to detect known as well as unexpected CNS pathogens (7–11). However, it is less clear whether mNGS has comparable sensitivity as compared with traditional pathogen-detection methods for diagnosing a specific CNS infection. Clinicians may still want to know whether pathogen-specific nucleic acid test is needed when an mNGS reveals a negative result for the pathogen of interesting. For the diagnosis of CNS *Mycobacterium tuberculosis* (MTB) infection, studies have shown that mNGS has higher sensitivity as compared with MTB-specific PCR (12, 13). Whether similar finding could be found in viral encephalitis needs to be further studied.

The sensitivity of CSF mNGS for pathogen detection may be greatly influenced by the CSF pleocytosis which leads to a substantial human DNA contamination (14). Since viral encephalitis generally has less inflamed CSF compared with other forms of CNS infections, the advantage of pathogen identification by mNGS may be more prominent in viral encephalitis. In the present study, we aimed to investigate the potency of CSF mNGS for identification of the etiologic agent using a model of varicella-zoster virus (VZV) CNS infection.

## Methods

### Patients

This study included 31 patients with suspected VZV CNS infection admitted to the second hospital of Nanjing, China, from August 2018 to November 2020. In Nanjing, the second hospital of Nanjing is the only designated hospital for managing patients with primary VZV infection. Patients with characteristic skin rashes suggestive of varicella and concomitant neurological symptoms were hospitalized for screening of possible VZV CNS involvement. Diagnostic procedures in routine clinical care for those patients included a cerebral MRI and analysis of CSF cell counts, chemistry, and bacterial culture. After obtained written informed consent, additional CSF samples were collected and stored at −80°C until analysis for VZV IgM antibodies, VZV PCR, and unbiased mNGS.

The medical records, such as epidemiological and demographic data, medical history, underlying comorbidities, clinical manifestations, laboratory tests, cerebral imaging findings, treatments, and outcomes, were retrieved from an electronic health record system. This study was approved by the ethics committee of the second hospital of Nanjing (reference number: 2020-LY-kt061).

### Metagenomic Next-Generation Sequencing

Cerebrospinal fluid samples were sent for PMSeq-DNA, a commercially available service from BGI-Shenzhen, which detects pathogenic microorganisms through mNGS. In this study, 600 μl CSF sample was mixed with enzyme and glass beads, and was vortexed vigorously at 2,800–3,200 rpm for 30 min. Then, total DNA was extracted from 300 μl each CSF sample using TIANamp Micro DNA Kit (DP316, Tiangen Biotech, Beijing, China) according to the recommendation of manufacturer. DNA libraries were constructed through DNA-fragmentation, end-repair, adapter-ligation, and PCR amplification. After quality control with Agilent 2100 (Agilent Technologies, CA, USA), libraries were sequenced by BGISEQ-50 platform. Low-quality sequence reads and sequence reads mapped to the human reference genome (hg19) were removed. The remaining data were classified by aligning to four Microbial Genome Databases containing bacteria, fungi, viruses, and parasites. The reference databases were downloaded from NCBI (ftp://ftp.ncbi.nlm.nih.gov/genomes/) which contained 4,945 whole genome sequences of viral taxa, 6,350 bacterial genomes or scaffolds, 1,064 pathogenic fungi genomes, and sequences of 234 parasites associated with human diseases.

### Detection of VZV-IgM Antibodies

An ELISA kit (ESR104M, SERION, Friedrich-Bergius-Ring, Würzburg, Germany) was used to measure VZV-IgM antibodies in CSF samples. The ELISA procedure was done following SERION ELISA classic CSF Diagnostics instructions with a modification that the dilution factor of CSF samples was changed from 1:20 to 1:10. As a CSF standard was not included in the kit, the interpretation of ELISA results was based on average optical density (OD) 450 values. The ELISA test was considered negative if the average OD value from duplicate sample wells is <0.1 or was <2-fold of that in the negative control. In our study, CSF samples from nine patients with syphilis-seropositive were used as negative control. Those nine patients were excluded from neurosyphilis based on normal CSF cell counts and chemistry tests, and negative CSF *treponema pallidum* particle agglutination assays. The ELISA assay was considered positive if the average OD value from each of the duplicate sample wells was ≥2-fold of that in the negative control.

### Real-Time PCR Detection Assay

The VZV nucleic acid was detected by real-time PCR assay. For the qualitative assay, 30 μl of total DNA was extracted from 200 μl CSF sample using TIANamp Micro DNA Kit (DP316, Tiangen Biotech). Then, the presence of VZV DNA was detected using a commercially available VZV nucleic acid detection kit following the instruction of manufacturer (D2211, Daan Gene, Guangdong, China). Each sample was testes two times. The following PCR program was run in the applied biosystems (ABI) 7500 Thermal Cycler (Applied Biosystems, MA, USA): 50°C for 2 min; 95°C for 15 min; 45 cycles of 94°C for 15 s; and 55°C for 45 s. A cycle threshold (Ct) value of 38 or less was considered positive.

In addition, the CSF samples were sent for absolute quantification of the VZV viral load using an in-house real-time quantification PCR test at a third-party diagnostic company (New Jingpei Diagnostics, Shanghai, China). Then, 100 μl of total DNA was extracted from 200 μl CSF sample using a DNA/RNA extraction Kit (ZTLJB, Tianlong Biotech, Hangzhou, China). Furthermore, 5 μl DNA sample was added to the reaction mix prepared following instruction of QuantiTect Probe PCR Kit (204343, Qiagen, Germany). The primers and probe set for VZV DNA amplification and detection were as follows: forward primer: 5′-TGCAGGGCATGGCTCAGT-3′; reverse primer: 5′-CCCAAGAACCACATGTCCAAC-3′; and probe: 5′-FAM-TCCTCAGTGCGGTGGTTGCCCA-BHQ1-3′ (15). The following PCR program was run in the applied biosystems (ABI) 7500 Thermal Cycler: 50°C for 2 min; 95°C for 15 min; 45 cycles of 94°C for 15 s; and 60°C for 40 s. Standard curve for absolute quantification was generated by amplifying serial dilutions of a plasmid containing VZV DNA segment. The lower limit of detection as evaluated by the service provider was 500 copies/ml.

### Statistical Analysis

Continuous variables were expressed as the medians and interquartile ranges (IQRs). Categorical variables were shown as the counts and percentages in each category. Comparison between groups was done using McNemar's test for categorical variables, when appropriate. Statistical analysis was done by using SPSS version 22.0 (IBM, NY, USA). A *p* < 0.05 is considered statistically significant.

## Results

### The Characteristics of the Patients

The characteristics of the 31 patients admitted for screening of the VZV CNS infection were summarized in Table 1. None of the patients previously received chickenpox vaccine or had any evidence of immune deficiency. The diagnosis of primary VZV infection was mainly based on the presence of typical rashes morphologically resembling chickenpox. Serum VZV IgM antibodies were positive in 66.7% (8/12) of the patients. The median age was 30.0 (IQR, 24.3–33.3) years. About 51.6% (16/31) of the patients were men, and 54.8% (17/31) patients had contacted with chickenpox within 4 weeks. The median time from onset of illness to admission was 4.0 (3.0–5.0) days, while from onset of neurological symptoms was 2.0 (1.0–4.0) days. The most common neurological symptom was headache (93.5%, 29/31), followed by nausea (45.2%, 14/31) and vomiting (19.4%, 6/31). Signs of meningeal irritation were not common, with nuchal rigidity seen in 12.9% (4/31) of the patients and Kernig's sign in 3.2% (1/31) of the patient. The majority of the patients (80.6%, 25/31) had CSF white blood cell counts not more than 5 × 10^6^ cells/L.

**Table 1:** Clinical, laboratory, and imaging characteristics of the 31 patients hospitalized for screening of varicella-zoster virus (VZV) central nervous system (CNS) infection.

	**Total (*n* = 31)**
**Demographics and clinical characteristics**	
Age, years	30 (24.3–33.3)
Sex, male	16 (51.6)
Comorbidity	2 (6.5)
Recently contacted with chickenpox	17 (54.8)
Time from chickenpox onset, days	4.0 (3.0–5.0)
Time from neurological symptoms onset, days	2 (1–4)
Headache	29 (93.5)
Neuasia	14 (45.2)
Vomitting	6 (19.4)
Mental Change	2 (6.5)
Difficulty urinating	2 (6.5)
Nuchal rigidity	4 (12.9)
Kernig's sign	1 (3.2)
Brudzin ski's sign.	0 (0)
**Blood laboratory tests**	
White blood cell count, × 10^9^/L	4.9 (3.9–7.0)
Lymphocyte count, × 10^9^/L	1.3 (1.1–2.9)
Lactate dehydrogenase, IU/L	240.0 (202.5–289.5)
C-reactive protein, mg/L	10.8 (2.7–20.9)
**Cerebrospinal fluid analysis**	
White blood cell count, × 10^6^/L	2.0 (1.0–5.0)
Proteins, mg/L	261.4 (219.7–354.8)
Glucose, mmol/L	3.2 (2.9–3.8)
Lactate dehydrogenase, IU/L	17 (14–21)
Adenosine deaminase	2 (0.1–3.6)
Negative bacterial culture	31 (100)
**Brain imaging**	
Abnormal magnetic resonance imaging	4 (12.9)

### Diagnosis of VZV CNS Infection Through CSF VZV IgM Detection, VZV PCR, and mNGS

At the time of discharge, 41.9% (13/31) of the patients were clinically diagnosed with VZV CNS infection. Of the 13 patients with a clinical diagnosis of VZV CNS infection, 6 had CSF pleocytosis (leukocytes >5 × 10^6^ cells/L). The diagnosis of VZV CNS infection in the remaining 7 patients with normal CSF white blood cell counts was made by expert penal after thoroughly evaluating the clinical course and imaging findings of those patients. To explore the microbiological evidence of VZV CNS infection, stored CSF samples were tested for VZV IgM antibodies, VZV PCR, and unbiased mNGS. VZV IgM antibodies were presented in 16.1% (5/31) of the CSF samples.

Varicella-zoster virus nucleic acids were detected in 16.1% (5/31) and 9.7% (3/31) of the CSF samples, respectively, when different real-time PCR reagents were used. VZV nucleic acids were detected in three CSF samples by both qualitative and quantitative VZV PCR. Two CSF samples, with negative quantitative VZV PCR results, tested weak positive for VZV by using qualitative VZV PCR with cycle threshold of 37.88 and 37.93, respectively. Intriguingly, maximal identification of VZV elements was achieved by CSF mNGS (*p* = 0.001 and *p* = 007; compared with qualitative PCR and VZV IgM antibody test, respectively), with sequence reads of VZV being reported in 51.6% (16/31) of the CSF samples (Table 2). The VZV relative abundance was provided in Supplementary Table 1. All the cases with positive CSF VZV PCR results tested positive for VZV sequence reads with CSF mNGS (Table 2).

**Table 2:** Diagnosis of VZV CNS infection.

	**CSF mNGS** ^**a**^	
	**VZV detected (*n* = 16)**	**VZV not detected (*n* = 15)**
Clinical diagnosed VZV CNS infection	7 (43.8)	6 (40.0%)
Positive CSF VZV IgM antibodies	3 (18.8)	2 (13.3)
CSF VZV nucleic acid test (qualitatively)	5 (31.5)	0 (0)
CSF VZV nucleic acid test (quantitatively)	3 (18.8)	0 (0)
Abnormal cerebral MRI	1 (6.3)	3 (20)

*CSF, cerebrospinal fluid; mNGS, metagenome next-generation sequencing; VZV, varicella-zoster virus; CNS, central nervous system; MRI, magnetic resonance imaging*.

a *The median [interquartile range (IQR)] sequence reads of CSF mNGS is 34.6 (28.8–48.3) millions*.

### Pathogens Unexpectedly Identified by mNGS and the Clinical Significance

**Figure 1:**
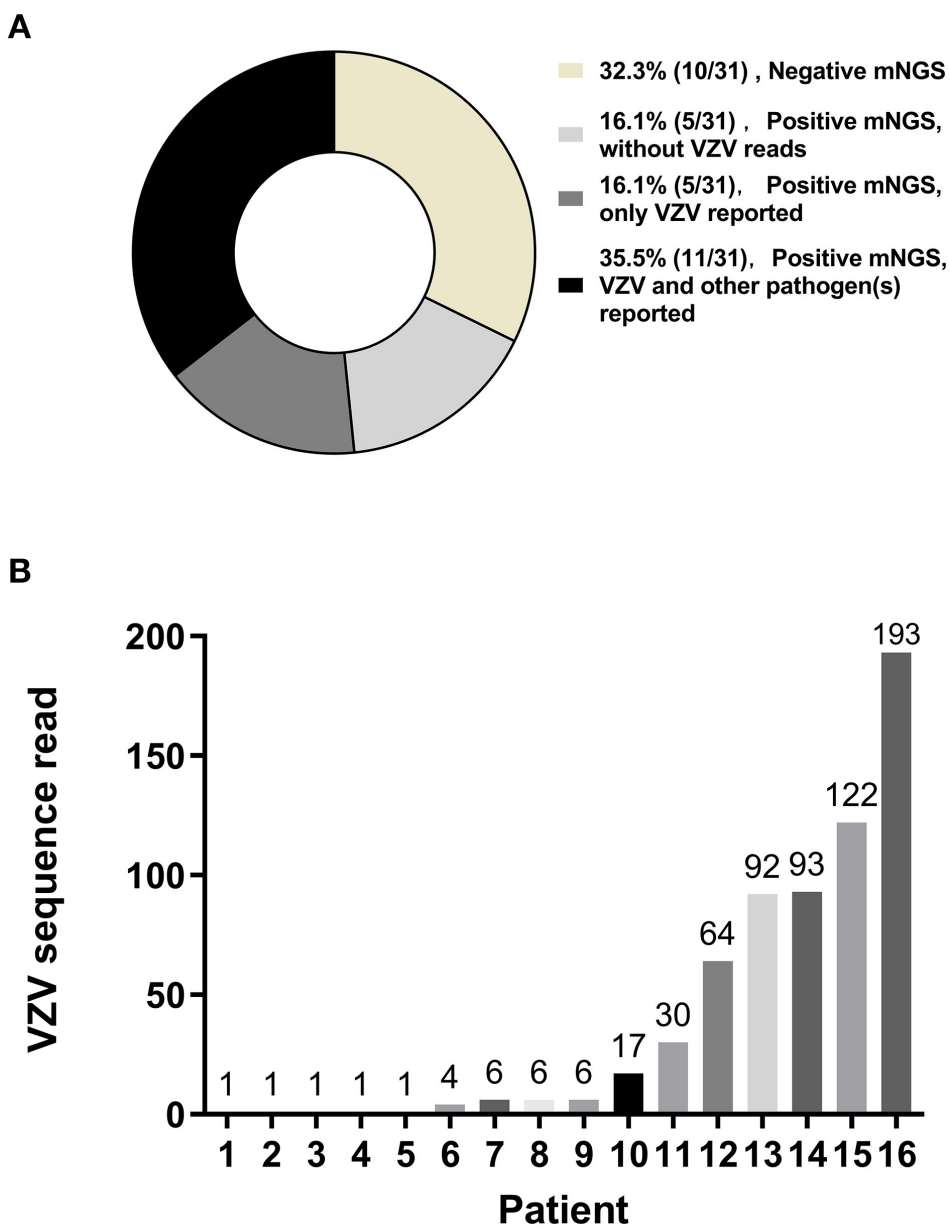
Metagenomic next-generation sequencing (mNGS) of cerebrospinal fluid (CSF) samples. Metagenomic next-generation sequencing was performed on CSF samples from 31 patients with suspected varicella-zoster virus (VZV) central nervous system (CNS) infections. VZV sequence reads were the only sequence reads reported in 16.1% (5/31) of the patients. VZV sequence reads and sequences reads of other pathogens were both reported in 35.5% (11/31) of the patients **(A)**. Of those 16 patients with VZV being detected by CSF mNGS, variable numbers of VZV sequence reads were reported **(B)**.

Of the 16 patients with VZV DNA detected by mNGS, the median VZV read was 6 (IQR, 1–85) (Figure 1A); VZV was the only reported pathogen in five CSF samples, and the remaining 11 CSF samples also detected other pathogens, most commonly the CMV (5 CSF samples; range of sequence reads, 1–26) (Figure 1B). Pathogens unexpectedly identified by mNGS in the 31 CSF samples were shown in Table 3; of which, 5 had HSV-1 sequence reads (range, 1–155). The presence of CMV and HSV-1 was evaluated by two real-time PCR based kits (Sansure Biotech, Hunan, China), and none of the CSF sample showed positive result.

**Table 3:** Pathogens unexpectedly reported by the mNGS.

	**No. of positive CSF sample**	**No. of sequence reads**
Cytomegalovirus	5	1, 4, 5, 7, and 26
Herpes simplex virus-1	5	1, 1, 4, 8, and 155
Human polyomavirus-5	2	3 and 3
*Candida albicans*	2	4 and 10
*Mycoplasma* species	2	3 and 5
Others: human betaherpesvirus 6A, human papillomavirus-5, *Staphylococcus aureus, Rickettsia felis, Aspergillus fumigatus*, Torque teno virus 28, human papillomavirus-19, Epstein-Barr virus, and molluscum contagiosum virus	1 for each pathogen	889, 10, 4, 4, 4, 3, 3, 2 and 2, respectively

*CSF, cerebrospinal fluid*.

A mNGS of the CSF sample revealed relatively high sequence reads of human betaherpesvirus 6A (HHV-6A) and low sequence reads of VZV (889 and 6, respectively). The patient was admitted to the hospital because of fever and itchy blisters for 6 days and difficulty urinating for 1 day. She had a close contact with her son who was recently diagnosed with chickenpox. CSF analysis demonstrated pleocytosis with leukocytes of 120 × 10^6^ cells/L. A cerebral MRI showed multiple abnormalities in left thalamus, cerebellar vermis, and left cerebral peduncles. At that time, she was clinically diagnosed with VZV CNS infection, and was treated with acyclovir, intravenous immunoglobulin, and dexamethasone. The clinical condition was gradually improved. After hospitalization for 26 days, the patient was discharged without obvious symptoms. The presence of HHV-6A in the stored CSF sample, which was unexpectedly identified by mNGS, was further confirmed by real-time PCR following the protocol described by Gautheret-Dejean et al. (16).

## Discussions

Viral encephalitis accounts for 20–50% of microbiologically confirmed cases of encephalitis, with herpes simplex virus being the most common etiologic agent followed by VZV (17). Detection of the specific viral IgM antibodies or nucleic acid in CSF sample is regarded as a confirmation of viral encephalitis (3, 17). As for the VZV encephalitis, those parameters have variably diagnostic sensitivities, possibly due to different patients being enrolled in the studies (18–21). In a study on VZV CNS infections, VZV nucleic acid was detectable in the CSF only in one-fourth of the patients (18). Since CSF VZV viral load is correlated with the severity of VZV CNS infection (22, 23), patients with mild neurological involvement may have extremely low viral burden below the detection limit of VZV PCR. The presence of VZV IgM antibody in CSF could be analyzed by using the commercial ELISA kits. However, a CSF standard is generally not provided by the manufacturers, which makes setting a cut-off value complicated. This may be one of the reasons that contributes to the low utility of this parameter in diagnosing VZV CNS infection, although a study revealed a low sensitivity of CSF IgM antibody for identification of VZV CNS infection (18).

For the above-mentioned reasons, a negative CSF VZV IgM test or nucleic acid test does not necessarily rule out VZV CNS infection. Therefore, in the present study, we did not arbitrarily define “true positive” or “true negative” cases of VZV CNS infection. In this context, the exact sensitivity of each microbiological test for diagnosing of CNS infection was not calculated. Instead, the magnitude of positive result was compared. Our study showed that, compared with CSF VZV IgM antibody test or CSF VZV PCR test, CSF mNGS identified much higher number of cases with clues of VZV CNS infection. Of note, all the CSF samples with positive VZV PCR results tested positive for VZV sequence reads by using mNGS. Those findings suggest that CSF VZV-specific PCR may not improve the sensitivity of diagnosing VZV CNS infection when mNGS has already been performed on CSF samples.

The mNGS focuses on the whole genome sequence of the pathogen which is broken into small sequence segments that are unbiasedly amplified and measured. Therefore, sequences of a given pathogen are more likely to be detected by mNGS targeting the whole genome of the pathogen, as compared with pathogen-specific PCR which generally targets single or two regions of the genome sequence (24). Moreover, in the case of target gene mutation, pathogen-specific PCR may not be able to amplify the target gene, which leads to a false negative result. The abovementioned reasons could explain why, in our study, mNGS performed better with regard to the detection of VZV sequences compared with PCR methods. Finally, as shown in Table 1, the level of CSF white blood cells infiltration is relatively low. In this condition, the sensitivity of mNGS for pathogen detection may be less impacted by human genome contamination.

The main advantage of mNGS is unbiased sampling which enables broad identification of pathogens in a hypothesis-free manner (6). Using this relatively novel strategy, others and we have already diagnosed a number of unexpected or rare cases with infectious etiologies (7, 9, 25–28). In the current study on patients with suspected VZV CNS infection, HHV-6A was unexpectedly identified by CSF mNGS from a patient with obvious neurological symptoms. Since HHV-6A has been shown to be related to neurological diseases (29–33), the presence of HHV-6A in the CSF sample of our patient could be of clinical importance. The finding re-emphasizes that agnostic mNGS is a useful strategy for identification of the etiologic agent when the treating physician is not familiar with the encountered clinical situation.

The mNGS is not a perfect diagnostic strategy in clinical microbiology. The unbiased sampling is not just an advantage but also, to some extent, could be a drawback. First, clinical samples are dominated by human host background. Unwanted human DNA occupies the majority of sequence reads of the mNGS which would limit the sensitivity of this strategy for pathogen identification (6, 34). Second, sequence reads from microorganisms not related to the clinical diseases can also be reported, which complicates the interpretation of the mNGS results. Those microbial contaminants, such as environmental microbes and skin flora, could be generated during sample collection and processing. Even the reagents used for processing may be contaminated by microbial DNA (6, 34). Finally, mNGS does not have the power to distinguish invasive infection from colonization. In addition, it could not differentiate between latent and active infections. In this condition, mNGS results should be interpreted based on the clinical judgement of the treating physicians.

As shown in Table 3, trace amounts of microbial nucleic acids, most commonly the CMV and HSV-1, were reported. CMV and HSV-1 are common human pathogens, both of which could cause CNS diseases (3). The mNGS, itself, could not answer whether those microbial DNAs really presented in the CSF samples. Additionally, it is not possible to tell whether those pathogens caused the neurological symptoms of our patients. Those drawbacks of mNGS are not likely to be completely resolved in the near future. At this point, it is advocated that mNGS results are treated as a list of candidate etiological agents which help to narrow the diagnosis. Only when the limitations of the mNGS are fully understood, then the clinicians may properly interpretate the mNGS results.

In conclusion, our study found that CSF mNGS was the most sensitive microbiological test for diagnosing VZV CNS infection, and could unexpectedly identify the causative agent that were not recognized based on traditional diagnostic tests. Perhaps, for the diagnoses of viral encephalitis caused by DNA virus, with continuing improving the methodology of mNGS, pathogen-specific PCR may not be needed when mNGS has already been used for pathogen detection. Nevertheless, pathogen-specific antibody detection may still be performed because it provides different biological information. Finally, although the sensitivity of mNGS can be further increased by technical innovation, the specificity will continue to be a great concern. Clinical judgement of the treating physicians is very important for interpretating the results.

## Data Availability Statement

The datasets used and/or analyzed during the current study are available from the corresponding author on reasonable request. The raw sequences of mNGS were deposited to the NCBI database under accession numbers PRJNA784638.

## Ethics Statement

The studies involving human participants were reviewed and approved by the Second Hospital of Nanjing. Written informed consent to participate in this study was provided by the participants' legal guardian/next of kin.

## Author Contributions

ZH designed this study. YZ, CD, and ZH collected the data. MX, WW, and ZH completed the experiment. MX, ZP, WC, and ZH analyzed the data. BS, JC, and CC were involved in the clinical management of the patients and analyzed the clinical data. This manuscript was initially drafted by YZ, CD, and ZH and then revised by MX, ZP, WW, BS, JC, CC, WC, HW, and ZH. All authors approved the final manuscript.

## Funding

This study was funded in part by the project of Jiangsu province medical youth talent (QNRC2016059) and the Nanjing medical science and technique development foundation (YKK18153). The funders had no role in study design, data collection and analysis, decision to publish, or preparation of the manuscript.

## Conflict of Interest

The authors declare that the research was conducted in the absence of any commercial or financial relationships that could be construed as a potential conflict of interest.

## Publisher's Note

All claims expressed in this article are solely those of the authors and do not necessarily represent those of their affiliated organizations, or those of the publisher, the editors and the reviewers. Any product that may be evaluated in this article, or claim that may be made by its manufacturer, is not guaranteed or endorsed by the publisher.

## References

[1] Somand D Meurer W. Central nervous system infections. Emerg Med Clin North Am. (2009) 27:89–100. 10.1016/j.emc.2008.07.004 19218021

[2] Dubot-Pérès A Mayxay M Phetsouvanh R Lee SJ Rattanavong S Vongsouvath M. Management of central nervous system infections, vientiane, laos, 2003-2011. Emerg Infect Dis. (2019) 25:898–910. 10.3201/eid2505.180914 31002063 PMC6478220

[3] Tunkel AR Glaser CA Bloch KC Sejvar JJ Marra CM Roos KL. The management of encephalitis: clinical practice guidelines by the Infectious Diseases Society of America. Clin Infect Dis. (2008) 47:303–27. 10.1086/589747 18582201

[4] Granerod J Cunningham R Zuckerman M Mutton K Davies NW Walsh AL. Causality in acute encephalitis: defining aetiologies. Epidemiol Infect. (2010) 138:783–800. 10.1017/S0950268810000725 20388231

[5] He T Kaplan S Kamboj M Tang YW. Laboratory diagnosis of central nervous system infection. Curr Infect Dis Rep. (2016) 18:35. 10.1007/s11908-016-0545-6 27686677 PMC5612431

[6] Gu W Miller S Chiu CY. Clinical metagenomic next-generation sequencing for pathogen detection. Ann Rev Pathol. (2019) 14:319–38. 10.1146/annurev-pathmechdis-012418-012751 30355154 PMC6345613

[7] Hu Z Weng X Xu C Lin Y Cheng C Wei H. Metagenomic next-generation sequencing as a diagnostic tool for toxoplasmic encephalitis. Ann Clin Microbiol Antimicrob. (2018) 17:45. 10.1186/s12941-018-0298-1 30587202 PMC6305995

[8] Wilson MR Sample HA Zorn KC Arevalo S Yu G Neuhaus J. Clinical metagenomic sequencing for diagnosis of meningitis and encephalitis. N Engl J Med. (2019) 380:2327–40. 10.1056/NEJMoa1803396 31189036 PMC6764751

[9] Fang M Weng X Chen L Chen Y Chi Y Chen W. Fulminant central nervous system varicella-zoster virus infection unexpectedly diagnosed by metagenomic next-generation sequencing in an HIV-infected patient: a case report. BMC Infect Dis. (2020) 20:159. 10.1186/s12879-020-4872-8 32075599 PMC7031966

[10] Hong NTT Anh NT Mai NTH Nghia HDT Nhu LNT Thanh TT. Performance of metagenomic next-generation sequencing for the diagnosis of viral meningoencephalitis in a resource-limited setting. Open Forum Inf Dis. (2020) 7:ofaa046. 10.1093/ofid/ofaa046 32158774 PMC7051036

[11] Xing X-W Zhang J-T Ma Y-B He M-W Yao G-E Wang W. Metagenomic next-generation sequencing for diagnosis of infectious encephalitis and meningitis: a large, prospective case series of 213 patients. Front Cell Infect Microbiol. (2020) 10:88. 10.3389/fcimb.2020.00088 32211343 PMC7066979

[12] Wang S Chen Y Wang D Wu Y Zhao D Zhang J. The feasibility of metagenomic next-generation sequencing to identify pathogens causing tuberculous meningitis in cerebrospinal fluid. Front Microbiol. (2019) 10:1993. 10.3389/fmicb.2019.01993 31551954 PMC6733977

[13] Yan L Sun W Lu Z Fan L. Metagenomic Next-Generation Sequencing (mNGS) in cerebrospinal fluid for rapid diagnosis of Tuberculosis meningitis in HIV-negative population. Int J Inf Dis. (2020) 96:270–5. 10.1016/j.ijid.2020.04.048 32339718

[14] Ji X-C Zhou L-F Li C-Y Shi Y-J Wu M-L Zhang Y. Reduction of human DNA contamination in clinical cerebrospinal fluid specimens improves the sensitivity of metagenomic next-generation sequencing. J Mol Neurosci. (2020) 70:659–66. 10.1007/s12031-019-01472-z 32002752

[15] Persson A Bergström T Lindh M Namvar L Studahl M. Varicella-zoster virus CNS disease–viral load, clinical manifestations and sequels. J Clin Virol. (2009) 46:249–53. 10.1016/j.jcv.2009.07.014 19709927

[16] Gautheret-Dejean A Manichanh C Thien-Ah-Koon F Fillet AM Mangeney N Vidaud M. Development of a real-time polymerase chain reaction assay for the diagnosis of human herpesvirus-6 infection and application to bone marrow transplant patients. J Virol Methods. (2002) 100:27–35. 10.1016/S0166-0934(01)00390-1 11742650

[17] Tyler KL. Acute viral encephalitis. N Engl J Med. (2018) 379:557–66. 10.1056/NEJMra1708714 30089069

[18] Koskiniemi M Piiparinen H Rantalaiho T Eränkö P Färkkilä M Räihä K. Acute central nervous system complications in varicella zoster virus infections. J Clin Virol. (2002) 25:293–301. 10.1016/S1386-6532(02)00020-3 12423693

[19] Gregoire SM Van Pesch V Goffette S Peeters A Sindic CJ. Polymerase chain reaction analysis and oligoclonal antibody in the cerebrospinal fluid from 34 patients with varicella-zoster virus infection of the nervous system. J Neurol Neurosurg Psychiatry. (2006) 77:938–42. 10.1136/jnnp.2006.090316 16844949 PMC2077607

[20] Nagel MA Cohrs RJ Mahalingam R Wellish MC Forghani B Schiller A. The varicella zoster virus vasculopathies: clinical, CSF, imaging, and virologic features. Neurology. (2008) 70:853–60. 10.1212/01.wnl.0000304747.38502.e8 18332343 PMC2938740

[21] De Broucker T Mailles A Chabrier S Morand P Stahl J-P. Acute varicella zoster encephalitis without evidence of primary vasculopathy in a case-series of 20 patients. Clin Microbiol Inf. (2012) 18:808–19. 10.1111/j.1469-0691.2011.03705.x 22085160

[22] Aberle SW Aberle JH Steininger C Puchhammer-Stöckl E. Quantitative real time PCR detection of Varicella-zoster virus DNA in cerebrospinal fluid in patients with neurological disease. Med Microbiol Immunol. (2005) 194:7–12. 10.1007/s00430-003-0202-1 14997388

[23] Rottenstreich A Oz ZK Oren I. Association between viral load of varicella zoster virus in cerebrospinal fluid and the clinical course of central nervous system infection. Diagn Microbiol Infect Dis. (2014) 79:174–7. 10.1016/j.diagmicrobio.2014.02.015 24666705

[24] Metzker ML. Sequencing technologies—the next generation. Nat Rev Genet. (2010) 11:31–46. 10.1038/nrg2626 19997069

[25] Mongkolrattanothai K Naccache SN Bender JM Samayoa E Pham E Yu G. Neurobrucellosis: unexpected answer from metagenomic next-generation sequencing. J Pediatric Infect Dis Soc. (2017) 6:393–8. 10.1093/jpids/piw066 28062553 PMC6251619

[26] Brown JR Bharucha T Breuer J. Encephalitis diagnosis using metagenomics: application of next generation sequencing for undiagnosed cases. J Infect. (2018) 76:225–40. 10.1016/j.jinf.2017.12.014 29305150 PMC7112567

[27] Huang Z Zhang C Li W Fang X Wang Q Xing L. Metagenomic next-generation sequencing contribution in identifying prosthetic joint infection due to Parvimonas micra: a case report. J Bone Joint Inf. (2019) 4:50–5. 10.7150/jbji.30615 30755848 PMC6367198

[28] Jin W Miao Q Wang M Zhang Y Ma Y Huang Y. A rare case of adrenal gland abscess due to anaerobes detected by metagenomic next-generation sequencing. Ann Transl Med. (2020) 8:123. 10.21037/atm.2020.01.123 32309394 PMC7154389

[29] Asano Y Yoshikawa T Kajita Y Ogura R Suga S Yazaki T. Fatal encephalitis/encephalopathy in primary human herpesvirus-6 infection. Arch Dis Child. (1992) 67:1484–5. 10.1136/adc.67.12.1484 1336954 PMC1793972

[30] Ogata M Satou T Kawano R Takakura S Goto K Ikewaki J. Correlations of HHV-6 viral load and plasma IL-6 concentration with HHV-6 encephalitis in allogeneic stem cell transplant recipients. Bone Marrow Transplant. (2010) 45:129–36. 10.1038/bmt.2009.116 19465942

[31] Ogata M Satou T Kadota J-I Saito N Yoshida T Okumura H. Human Herpesvirus 6 (HHV-6) reactivation and HHV-6 encephalitis after allogeneic hematopoietic cell transplantation: a multicenter, prospective study. Clin Infect Dis. (2013) 57:671–81. 10.1093/cid/cit358 23723198

[32] Leibovitch EC Jacobson S. Evidence linking HHV-6 with multiple sclerosis: an update. Curr Opin Virol. (2014) 9:127–33. 10.1016/j.coviro.2014.09.016 25462444 PMC4269240

[33] Eliassen E Hemond CC Santoro JD. HHV-6-associated neurological disease in children: epidemiologic, clinical, diagnostic, and treatment considerations. Pediatr Neurol. (2020) 105:10–20. 10.1016/j.pediatrneurol.2019.10.004 31932119

[34] Simner PJ Miller S Carroll KC. Understanding the promises and hurdles of metagenomic next-generation sequencing as a diagnostic tool for infectious diseases. Clin Infect Dis. (2018) 66:778–88. 10.1093/cid/cix881 29040428 PMC7108102

