# Supplementary material for: Metagenomic Next-Generation Sequencing vs. Traditional Microbiological Tests for Diagnosing Varicella-Zoster Virus Central Nervous System Infection
Source: Front Public Health. 2022 Jan 21;9:738412. doi: 10.3389/fpubh.2021.738412 (PMC8814106; doi:10.3389/fpubh.2021.738412)
Supplement: Supplementary file 1 [file Table_1.docx]

**Supplemental Table 1 VZV relative abundance**

| **Patient ID** | **VZV reads** | **VZV relative abundance1，%** |
| --- | --- | --- |
| **1** | **1** | **50** |
| **2** | **1** | **0.33** |
| **3** | **1** | **2.33** |
| **4** | **1** | **4.44** |
| **5** | **1** | **100** |
| **6** | **4** | **57.14** |
| **7** | **6** | **100** |
| **8** | **6** | **13.04** |
| **9** | **6** | **0.5** |
| **10** | **17** | **24.06** |
| **11** | **30** | **6.98** |
| **12** | **64** | **94.34** |
| **13** | **92** | **81.73** |
| **14** | **93** | **100** |
| **15** | **122** | **99.54** |
| **16** | **193** | **100** |

VZV relative abundance is defined as the proportion of VZV in the virus group.
